# A Brainstem Radiomics Framework to Distinguish Progressive Supranuclear Palsy from Parkinson's Disease

**DOI:** 10.1002/mds.70334

**Published:** 2026-05-01

**Authors:** Chiara Camastra, Jolanda Buonocore, Antonio Augimeri, Camilla Calomino, Maria Giovanna Bianco, Alessia Sarica, Pier Paolo Arcuri, Aldo Quattrone, Andrea Quattrone

**Affiliations:** ^1^ Neuroscience Research Center Magna Graecia University Catanzaro Italy; ^2^ Brain Health Imaging Centre Centre for Addiction and Mental Health (CAMH) Toronto Canada; ^3^ Biotecnomed S.C.aR.L. Catanzaro Italy; ^4^ Azienda Ospedaliero‐Universitaria Renato Dulbecco Catanzaro Italy; ^5^ Institute of Radiology Azienda Ospedaliero‐Universitaria Renato Dulbecco Catanzaro Italy; ^6^ Institute of Neurology, Department of Medical and Surgical Sciences Magna Graecia University Catanzaro Italy

**Keywords:** brainstem, machine learning, Parkinson's disease, progressive supranuclear palsy, radiomics

## Abstract

**Background:**

Differentiating progressive supranuclear palsy (PSP) from Parkinson's disease (PD) can be clinically challenging. In the neuroimaging field, radiomics has emerged as a promising approach to capture subtle microstructural and textural image alterations, improving differential diagnoses.

**Objective:**

To assess the diagnostic value of brainstem radiomic features from T1‐weighted magnetic resonance imaging (MRI) in distinguishing PSP from PD patients.

**Methods:**

This study included 433 participants from two independent cohorts: an Italian training cohort (84 PSP and 177 PD) and an international validation cohort (68 PSP and 104 PD). Radiomic features including first‐order, shape, and texture descriptors were extracted with PyRadiomics from brainstem segmentations generated by the automated deep‐learning‐based AssemblyNet pipeline. Classification models (Decision Tree, Support Vector Machine, Random Forest, and XGBoost) were trained using nested cross‐validation and tested on the independent cohort. Model interpretability was examined with SHapley Additive exPlanations.

**Results:**

Radiomics‐based models yielded high and consistent performance in distinguishing PSP from PD, higher than brainstem volume. In the validation cohort, Random Forest and XGBoost achieved the best performance (area under the curve [AUC]: 0.93 and 0.94, respectively). Texture‐ and intensity‐based radiomic features emerged as the most informative predictors, while shape descriptors showed lower relevance in discrimination between PSP and PD.

**Conclusions:**

Brainstem radiomics extracted from routine T1‐weighted MRI demonstrated excellent classification performance in distinguishing PSP from PD patients and generalized robustly across independent datasets. Texture‐based features captured microstructural disorganization not reflected by automated volumetry, underscoring the added value of radiomics for differential diagnosis in atypical parkinsonism and for integration in future multimodal biomarker frameworks. © 2026 The Author(s). *Movement Disorders* published by Wiley Periodicals LLC on behalf of International Parkinson and Movement Disorder Society.

Parkinson's disease (PD) and progressive supranuclear palsy (PSP) are two distinct neurodegenerative disorders within the parkinsonian spectrum, characterized by overlapping motor features but divergent pathophysiological substrates, progression rates, and treatment responses.^1^ PD primarily involves degeneration of dopaminergic neurons in the substantia nigra pars compacta^2^, ^3^ with deposition of alpha‐synuclein and Lewy body formation, whereas PSP is defined by four‐repeat (4R)‐tau protein accumulation and widespread neuronal loss, particularly in the brainstem and basal ganglia.^4^, ^5^, ^6^ Although clinical diagnostic criteria have improved over the years,^7^ misdiagnosis remains common, especially in early disease stages, with PSP frequently misclassified as PD due to overlapping motor signs and late appearance of vertical supranuclear gaze palsy.^8^, ^9^, ^10^ Conventional magnetic resonance imaging (MRI) can support the differential diagnosis through visual inspection of disease‐specific atrophy patterns, but it often lacks sensitivity.^10^, ^11^, ^12^ Over recent years, several brainstem‐based linear and planimetric measures have been proposed to support the differential diagnosis between PSP and PD,^13^, ^14^, ^15^, ^16^, ^17^, ^18^ overcoming the limitations of visual inspection. These measures showed consistent high accuracy across studies,^13^, ^19^, ^20^, ^21^, ^22^ but are typically performed manually, requiring dedicated expertise, and showing variability across centers and raters. Among automated brainstem‐based metrics, midbrain or brainstem volumetry have been the most widely investigated measures. While these volumetric measures have been successfully employed as progression biomarkers in clinical trials,^23^, ^24^, ^25^ they showed lower accuracy than planimetric or linear measures when used as a diagnostic biomarkers to support PSP diagnosis.^26^

Radiomics provides automatically extracted, high‐dimensional quantitative features that capture intensity, shape, and texture patterns beyond visual assessment or volumetry.^27^, ^28^, ^29^, ^30^, ^31^, ^32^ Originally developed and extensively applied in oncology,^33^ radiomics enables objective and reproducible characterization of tissue heterogeneity and has recently shown great promise in neurodegenerative disorders, including PD and atypical parkinsonian syndromes.^34^, ^35^ Several studies have demonstrated that radiomic features derived from nigral and striatal regions on quantitative susceptibility mapping (QSM), susceptibility‐weighted imaging (SWI), or T2‐weighted MRI can discriminate PD from healthy controls (HC) or from multiple system atrophy (MSA), often outperforming conventional imaging markers.^36^, ^37^, ^38^ However, most MRI‐based radiomic studies to date have focused on distinguishing PD from HC or MSA,^39^, ^40^ with fewer efforts aimed at differentiating between PSP and PD patients. In this study, we focused on the brainstem, a key region involved in both diseases and one of the earliest sites affected in the neurodegenerative process,^6^, ^17^, ^41^ aiming to distinguish PSP from PD patients in two large, independent patient cohorts.

## Patients and Methods

### Study Population

A total of 433 participants were included in this study, divided into a training cohort and an independent validation cohort. The training cohort comprised 177 patients with PD and 84 patients with PSP, recruited at the University “Magna Graecia” of Catanzaro, Italy. Clinical diagnoses were established by senior movement disorder specialists based on the Movement Disorder Society (MDS) clinical diagnostic criteria for progressive supranuclear palsy‐Richardson syndrome (PSP‐RS) and for PD.^7^, ^42^ All patients underwent a neurological examination off‐medication overnight, including the Movement Disorder Society‐revised Unified Parkinson's Disease Rating Scale‐Part‐III (MDS‐UPDRS‐III)^43^ and the Hoehn‐Yahr (HY) stage, and the Progressive Supranuclear Palsy Rating Scale (PSPRS) rating scale^44^ in PSP patients. All patients also underwent a brain 3 T MRI. The study was approved by the local ethics committee, and written informed consent was obtained from all participants for the use of their medical records for research purposes, in accordance with the Declaration of Helsinki.

The independent validation cohort included 104 PD and 68 PSP patients with available 3 T volumetric T1‐weighted brain MRI. PSP participants were obtained from the 4‐Repeat Tauopathy Neuroimaging Initiative (4RTNI) database (http://4rtni-ftldni.ini.usc.edu/). 4RTNI was launched in early 2011 and is funded through the National Institute of Aging and The Tau Research Consortium. The primary goal of 4RTNI is to identify neuroimaging and biomarker indicators for disease progression in the 4R tauopathy neurodegenerative diseases, PSP, and cortico‐basal degeneration (CBD). PSP patients were diagnosed according to the possible or probable National Institute of Neurological Disorders and Stroke and Society for PSP (NINDS‐SPSP) criteria. Available clinical data included age, sex, age at onset, disease duration and PSPRS rating scale. The data are the result of collaborative efforts in North America (https://clinicaltrials.gov/study/NCT01804452). All available data from 4RTNI PSP participants at baseline were selected, resulting in 72 PSP. Among these subjects, 4 PSP patients had no available MRI; thus, 68 PSP patients were included in the study. For more information on 4RTNI visit: http://memory.ucsf.edu/research/studies/4rtni. PD participants with age and disease duration similar to the training cohort were obtained from the Parkinson's Progression Markers Initiative (PPMI) database. Data used in the preparation of this article were obtained on May 2, 2025 from the PPMI database (www.ppmi‐info.org/access‐data‐specimens/download‐data), RRID: SCR_006431. For up‐to‐date information on the PPMI study visit www.ppmi-info.org. Ethics approval was obtained at each site from the local ethics committee, and all participants gave written informed consent. For clarity, throughout this article the cohort recruited at the University “Magna Graecia” is referred to as the training cohort, and the multicenter cohort derived from the PPMI and 4RTNI initiatives is referred to as the validation cohort.

### Image Preprocessing and Feature Extraction

All patients underwent a brain 3 T MRI examination including a three‐dimensional (3D) T1‐weighted sequence. Acquisition protocols are described in Supporting Information S1. The brainstem was segmented on the T1‐weighted MRI scans using two well‐established and validated automated segmentation pipelines: AssemblyNet^45^ and FreeSurfer.^46^ AssemblyNet is a deep learning‐based brain MRI segmentation framework that employs an ensemble of convolutional neural networks to delineate anatomical brain regions. For each subject, the segmentation outputs were saved as individual label maps. All FreeSurfer‐ and AssemblyNet‐derived segmentations underwent visual quality control to ensure correct alignment with the native T1‐weighted images and anatomical plausibility. All segmentations passed quality control, except for one PSP case in the training cohort, which showed a clear segmentation failure due to incomplete DICOM images and was therefore excluded from further analyses (Fig. S1). In parallel, all scans were processed using the FreeSurfer image analysis suite (version 7.4.1), following the standard recon‐all pipeline. This fully automated workflow performs cortical reconstruction and volumetric segmentation based on probabilistic atlases. For each participant, the brainstem mask was then used to extract radiomic features with the open‐source Python library PyRadiomics,^47^ following the Image Biomarker Standardization Initiative (IBSI) guidelines^48^ to ensure reproducibility and consistency. In total, 107 radiomic features were extracted, including 18 first‐order statistical and 14 shape‐based features, as well as a broad set of texture features: 24 derived from the gray‐level co‐occurrence matrix (GLCM), 16 from the gray‐level run length matrix (GLRLM), 16 from the gray‐level size zone matrix (GLSZM), 14 from the gray‐level dependence matrix (GLDM), and 5 from the neighboring gray tone difference matrix (NGTDM). The same feature extraction procedure was applied to brainstem masks obtained from both AssemblyNet and FreeSurfer segmentations. A detailed description of all radiomic feature classes and their mathematical definitions is available in the official PyRadiomics documentation (https://pyradiomics.readthedocs.io).

### Brainstem Volume Classification Performance

In this study, we first performed a classification analysis based on the brainstem volume in differentiating PSP from PD, using receiver operating characteristic (ROC) curve analysis. A set of candidate classification thresholds was derived from the ROC curve, and for each threshold, the associated Youden's J statistic (sensitivity + specificity −1),^49^ along with performance metrics including accuracy, sensitivity, specificity, precision, and F1‐score, were computed. The threshold that maximized Youden's J within the training cohort was selected as the fixed operating point for subsequent validation. This optimal threshold was then applied to the independent validation cohort to derive the corresponding classification metrics in the new dataset.

### Radiomics‐Based Machine Learning Classification Performance

Subsequently, brainstem radiomic features were used as input for machine learning classification aimed at distinguishing PSP from PD. Four well‐known supervised algorithms were compared, including Decision Tree,^50^ Support Vector Machine (SVM),^51^ Random Forest,^52^ and eXtreme Gradient Boosting (XGBoost).^53^ To reduce feature redundancy and mitigate overfitting, a two‐step unsupervised feature selection procedure not based on diagnostic labels was applied prior to model training. First, near‐constant features were removed using a variance threshold filter (variance <1 × 10^−8^). Second, highly correlated features were excluded using a Pearson correlation‐based filter (absolute r ≥ 0.95).^54^ The same reduced feature set was used as input for all four classifiers, ensuring a fair comparison. Feature standardization was incorporated into the learning pipeline to ensure comparability across feature scales.^55^ Model performance was assessed through a nested cross‐validation scheme with five outer and three inner folds.^56^ Within the inner loop, hyperparameters were optimized using grid search,^57^ while the outer loop provided unbiased performance estimates on unseen data. For each fold, multiple performance metrics were computed, including accuracy, precision, recall, specificity, F1‐score, and the area under the curve (AUC). For each classifier, predicted probabilities from the training folds were used to derive the optimal classification threshold by maximizing Youden's J statistic. The trained model was then frozen and directly applied to the independent validation cohort. All analyses were performed separately using radiomic features extracted from brainstem masks obtained from FreeSurfer and AssemblyNet. To assess the robustness of the proposed framework to different feature‐selection parameter settings, a sensitivity analysis was performed by varying the Pearson correlation threshold of the correlation‐based filter across five values (|r| ∈ {0.98, 0.95, 0.90, 0.85, 0.80}), leading to a decreasing number of features. For each scenario, model AUCs and performance degradation (|delta_AUC between training and testing|) were calculated. To gain insights into model interpretability, SHapley Additive exPlanations (SHAP)^58^ was applied to the best classifier, allowing assessment of the global contribution of individual radiomic features to the classification task. To further evaluate the reliability of the predicted probabilities, calibration analysis was performed for all classifiers. Calibration plots were generated by comparing the mean predicted probability with the observed fraction of positive outcomes across probability bins, with the diagonal representing perfect calibration. In addition, calibration performance was quantitatively assessed using the Brier score, which measures the mean squared error between predicted probabilities and observed outcomes, and the Hosmer–Lemeshow goodness‐of‐fit test, which evaluates agreement between predicted and observed event rates across probability bins. Finally, to evaluate the robustness of the classification pipeline against potential cohort‐specific confounds arising from the use of two separate international initiatives for the validation cohort (4RTNI for PSP and PPMI for PD), a reversed‐cohort analysis was performed. In this analysis, models were trained on the pooled PPMI and 4RTNI data and validated on the independent cohort from our site.

## Results

The training cohort comprised 177 patients with PD and 84 with PSP, whereas the validation cohort included 104 PD and 68 PSP patients (Table 1). Within each cohort, PSP and PD showed the expected clinical profile, with PSP patients being slightly older and more motor‐impaired than PD patients. Demographic and clinical characteristics were largely comparable between the two cohorts for both diagnostic groups, and the pattern of PSP–PD differences was similar in the training and validation sets, with the only notable discrepancy being a shorter disease duration in PSP patients in the training cohort compared with those in the validation cohort (Table 1).

**TABLE 1:** Demographic and clinical characteristics of the training and validation cohorts

	Training cohort			Validation cohort		
Characteristic	PD (*N* = 177)	PSP (*N* = 84)	*P*‐value	PD (*N* = 104)	PSP (*N* = 68)	*P*‐value
Sex (M/F)	116/61	43/41	0.039^a^	64/40	30/38	0.033^a^
Age at examination, years^b^	67.4 ± 7.7	69.7 ± 7.0	0.022^a^	66.4 ± 8.2	70.8 ± 7.5	<0.001^c*^
Disease duration, years^b^	4.6 ± 4.1	2.6 ± 1.3	< 0.001^c*^	4.2 ± 2.3	5.5 ± 3.9	0.016^c^
MDS‐UPDRS‐III^b^	23.8 ± 12.3	41.6 ± 15.7	< 0.001^c*^	27.9 ± 12.2	33.1 ± 17.2	0.030^c^
PSPRS^b^	–	39.8 ± 18.8	**–**		38.2 ± 16.8	**–**

*Note*: Clinical scores were available for: MDS‐UPDRS‐III (PD: 168/177 in training cohort, all in the validation cohort; PSP: 73/84 in training, 56/68 in validation) and PSPRS (PSP: 25/84 in training, 55/68 in validation).

^a^ Fisher's exact test.

^b^ Data are expressed as mean ± standard deviation.

^c^ *T*‐test or Wilcoxon rank sum test.

Abbreviations: PD, Parkinson's disease; PSP, progressive supranuclear palsy; M, male; F, female; MDS‐UPDRS‐III, Movement Disorders Society‐Unified Parkinson's Disease Rating Scale‐Part III; PSPRS, Progressive Supranuclear Palsy Rating Scale.

### Classification Performances

**FIG. 1:**
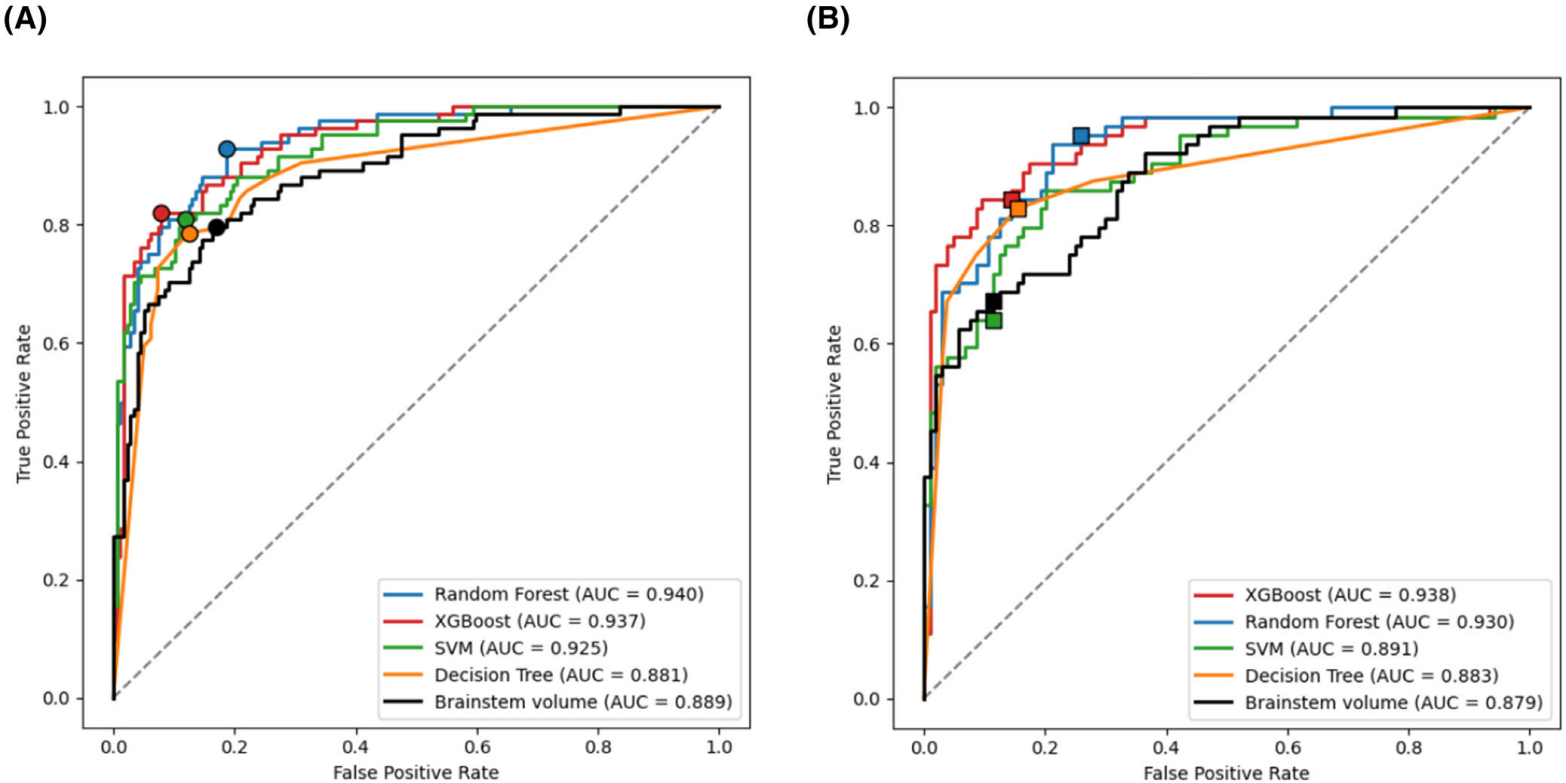
Receiver operating characteristic (ROC) curves for brainstem‐based classification of progressive supranuclear palsy versus Parkinson's disease using AssemblyNet‐derived features, showing the performance of radiomics models (XGBoost, Random Forest, Support Vector Machine (SVM), Decision Tree) compared with brainstem volume in the (A) training and (B) validation cohorts. Squares indicate the operating points corresponding to the maximal Youden index identified in the training cohort. Circles indicate the corresponding performance obtained in the validation cohort using the same fixed classification threshold. AUC, area under the curve. [Color figure can be viewed at wileyonlinelibrary.com]

Classification based on brainstem volume achieved fair AUC of 0.88–0.89 but barely acceptable accuracy of 81%–82% in the two cohorts. Machine learning models based on brainstem radiomic features showed higher performance in distinguishing PSP from PD compared with volume‐based analysis (Fig. 1). Results obtained using AssemblyNet‐derived features are summarized in Table 2. Among the different algorithms, XGBoost achieved the highest performance, with 89% accuracy and 0.94 AUC in the training cohort, and 88% accuracy and 0.94 AUC in the validation cohort. The Random Forest model yielded similar results, with 88% accuracy and 0.94 AUC in the training cohort and 85% accuracy and 0.93 AUC in the validation cohort (Fig. 1). For FreeSurfer‐derived radiomic features, classification performance was slightly lower overall but remained consistent across models. Random Forest and XGBoost again achieved the highest performance, with AUCs of 0.90 and 0.94 in the validation cohort, respectively (Table S1). To further illustrate the agreement between segmentation pipelines, we examined the subjects showing the largest volumetric discrepancies between AssemblyNet and FreeSurfer brainstem segmentations (Fig. S2). Importantly, all these subjects were correctly classified when using either AssemblyNet‐derived or FreeSurfer‐derived brainstem volumes, indicating that the observed volumetric differences did not affect the diagnostic classification. The slightly larger volumes obtained with FreeSurfer likely reflect systematic differences between the segmentation algorithms rather than differences that would influence the classification results. Model interpretability analysis based on SHAP highlighted the most influential radiomic features contributing to classification using FreeSurfer‐derived features (Fig. S3). A reversed‐cohort analysis was performed to evaluate potential cohort‐specific effects arising from the use of separate international initiatives for the validation dataset. When the models were trained on the pooled PPMI and 4RTNI data and validated on the independent cohort from our site, classification performance remained comparable to the primary analysis (validation AUCs of 0.93 and 0.94 for Random Forest and XGBoost, respectively), indicating that the proposed framework was not substantially influenced by bias in cohort composition or MRI protocols. The most important features identified in this reversed‐cohort setting are reported in Figure S4.

**TABLE 2:** Volume‐based and radiomics‐based classification performance using AssemblyNet segmentation

Algorithm	Accuracy (train/val)	AUC (train/val)	Precision (train/val)	Recall or sensitivity (train/val)	Specificity (train/val)	F1 (train/val)
Brainstem volume	0.82/0.81	0.89/0.88	0.69/0.80	0.79/0.69	0.83/0.88	0.74/0.72
Decision Tree	0.84/0.84	0.88/0.88	0.80/0.77	0.71/0.83	0.89/0.85	0.74/0.77
SVM	0.82/0.76	0.93/0.89	0.91/0.63	0.52/0.86	0.96/0.69	0.60/0.73
Random Forest	0.88/0.85	0.94/0.93	0.89/0.82	0.73/0.78	0.96/0.89	0.80/0.80
XGBoost	0.89/0.88	0.94/0.94	0.89/0.86	0.74/0.80	0.96/0.92	0.80/0.83

Abbreviations: train, training cohort; val, validation cohort; AUC, area under the curve; SVM, Support Vector Machine; XGBoost, eXtreme Gradient Boosting.

### Exploring the Contribution of Different Radiomics

**FIG. 2:**
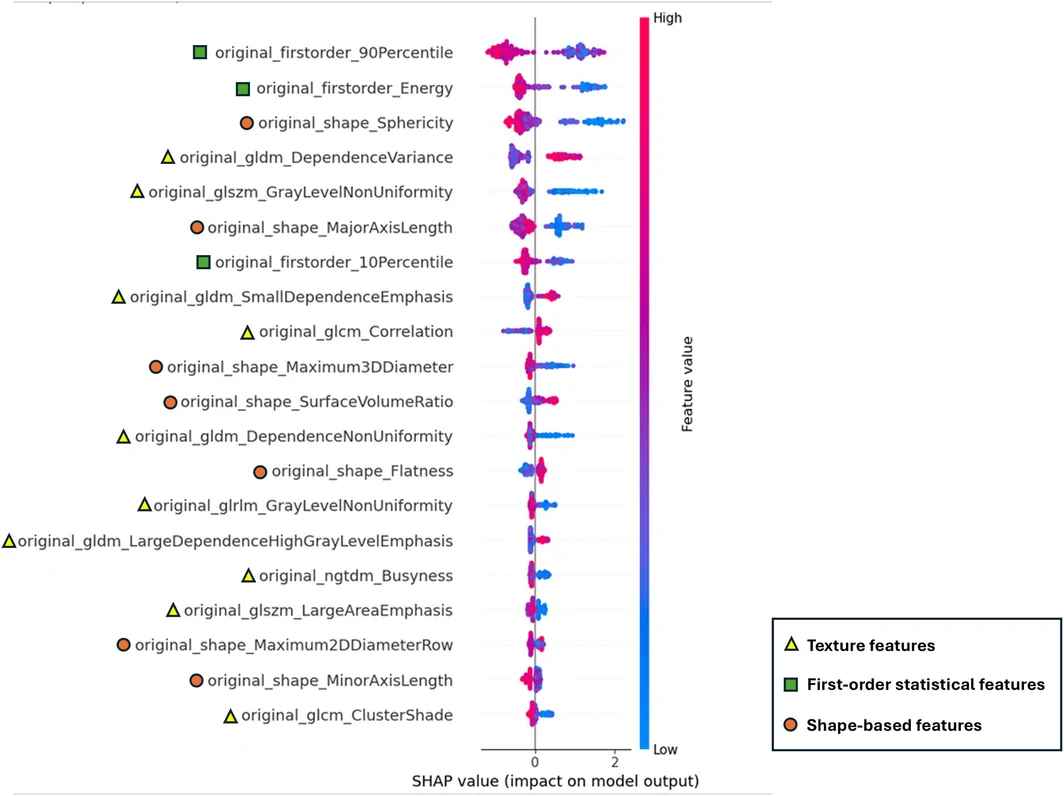
Global SHapley Additive exPlanations (SHAP) summary plot showing the contribution of each radiomic feature to the Support Vector Machine (SVM) model trained on AssemblyNet‐derived brainstem features. Each point represents one patient, with color indicating the feature value (red = high, blue = low). Features are ranked by mean absolute SHAP value, reflecting their overall impact on model output. [Color figure can be viewed at wileyonlinelibrary.com]

**FIG. 3:**
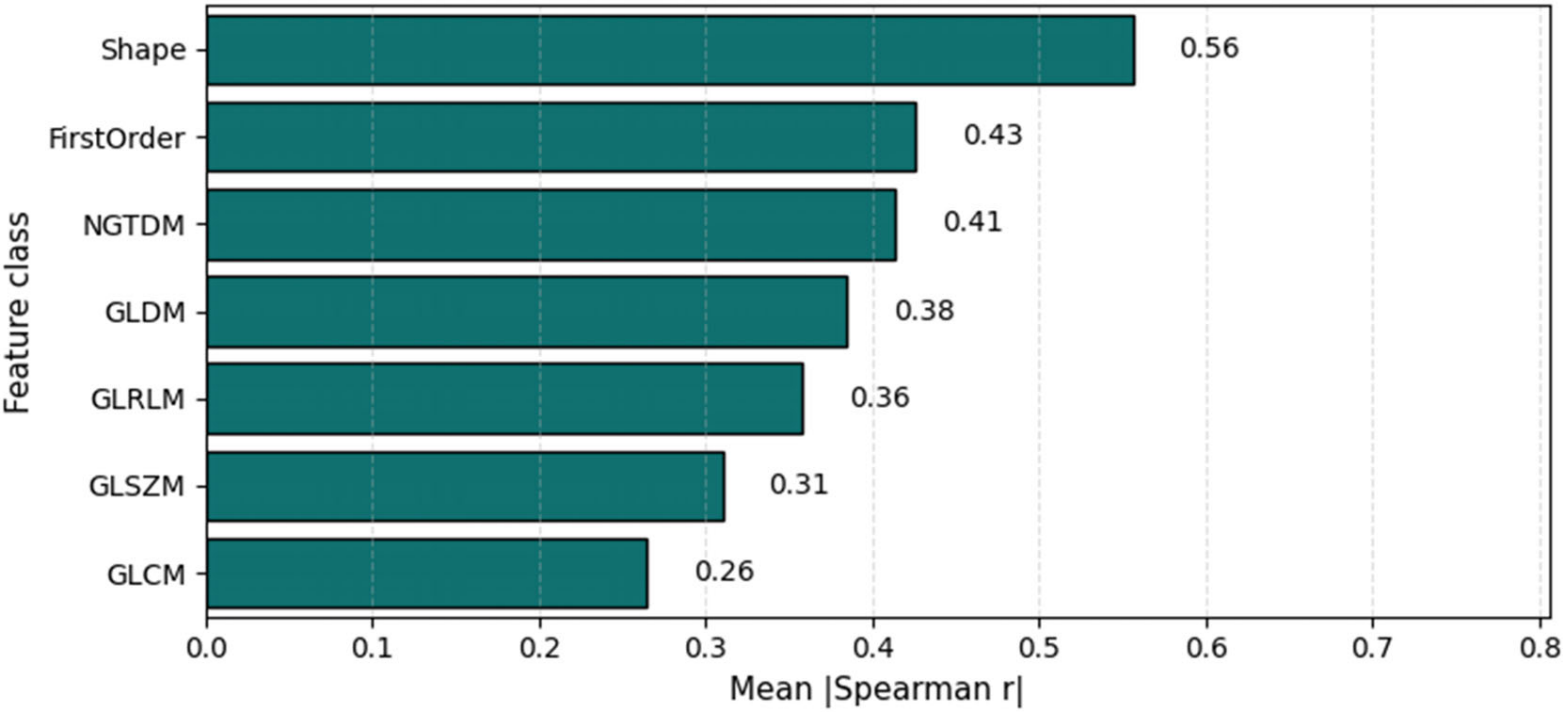
Correlation between brainstem volume and radiomic feature classes. The bars represent the mean Spearman's rank correlation coefficients between the brainstem volume and the individual extracted radiomic features of each category: shape, first‐order, neighboring gray tone difference matrix (NGTDM), gray‐level dependence matrix (GLDM), gray‐level run length matrix (GLRLM), gray‐level size zone matrix (GLSZM), and gray‐level co‐occurrence matrix (GLCM). [Color figure can be viewed at wileyonlinelibrary.com]

The unsupervised feature selection procedure retained 73 of the original 107 radiomic features, which were subsequently used as input for all classifiers. A sensitivity analysis using variable number of features demonstrated that XGBoost and Random Forest showed consistently high validation AUC and low performance degradation across all tested thresholds (|delta_AUC| ≤ 0.02 for Random Forest and XGBoost), confirming that the proposed framework is robust to variations in the feature‐selection parameter (Fig. S5). Model interpretability was further investigated using SHAP analysis applied to the best‐performing classifier (XGBoost trained on AssemblyNet‐derived features), as illustrated by the global SHAP summary plot (Fig. 2). The analysis revealed several texture‐ and intensity‐based features—such as first‐order 90th and 10th percentile, GLSZM and GLRLM gray‐level non‐uniformity, and GLDM dependence variance—ranked among the top contributors to model output. To further investigate whether the discriminative information captured by the SHAP‐identified feature classes was driven by morphometric size, we computed Spearman's rank correlations between brainstem volume and all extracted radiomic features, grouped by category (shape, first‐order, GLCM, GLRLM, GLSZM, GLDM, and NGTDM) as shown in Figure 3. As expected, shape features showed the highest correlation, whereas texture‐based classes (GLDM, GLRLM, GLSZM, NGTDM) exhibited weaker associations. Overall, several top‐ranked SHAP features were not significantly correlated with volume, suggesting that their discriminative power was not driven by morphometric size and may therefore complement volumetric information. Calibration analysis showed Brier scores <0.2 across classifiers, with the lowest value for XGBoost. The Hosmer–Lemeshow test did not indicate significant miscalibration (all *P* > 0.05), confirming good agreement between predicted and observed probabilities (Fig. S6).

## Discussion

In this study, we developed and applied a radiomics pipeline to systematically assess whether quantitative features extracted from the brainstem could distinguish PSP from PD, integrating high‐resolution T1‐weighted MRI, automated deep learning segmentation, and supervised machine learning models. The analyses revealed that radiomic features extracted from the brainstem yielded superior classification performance compared with conventional volumetric measures, achieving AUC values up to 0.93–0.94 in the validation cohort using well‐known decision tree‐based algorithms. These findings underscore the diagnostic value of radiomics in capturing microstructural tissue patterns that may be imperceptible to visual inspection yet highly informative for disease classification.

To date, most neuroimaging studies investigating radiomics in parkinsonian syndromes have focused on distinguishing PD from MSA or HC based on putamen or substantia nigra radiomics, often reporting strong classification accuracies using QSM, SWI, or T2‐weighted images, but rarely including PSP or performing external validation.^38^, ^59^, ^60^, ^61^, ^62^ Our study is, to our knowledge, the first to apply a radiomics‐based classification framework centered on the brainstem to distinguish PSP from PD, thus addressing an important gap in the current neuroimaging literature. We demonstrated that even using conventional T1‐weighted structural MRI, radiomics can reveal discriminative signatures potentially outperforming volumetry. The inclusion of both shape and texture‐based radiomic features enabled us to interrogate complementary pathological substrates, with texture features—particularly those derived from the gray‐level co‐occurrence matrix (GLCM), size zone matrix (GLSZM), and dependence matrix (GLDM)—emerging as dominant contributors in SHAP analysis. This pattern is consistent with prior studies reporting that PSP pathology produces widespread microstructural disorganization in the brainstem, which may be captured as signal variability on structural MRI sequences.^63^ To determine whether these radiomic features primarily reflected brainstem volume or rather captured complementary information, we conducted correlation analyses between each radiomic feature group and the brainstem volume. Most high‐ranking SHAP features belonged to radiomic groups showing only weak associations with brainstem volume, suggesting that these metrics encode distinct biological information and provided discriminative information not purely reflecting the structure volume. Overall, the SHAP summary plot (Fig. 2) revealed that the most relevant radiomic features reflected signal intensity, magnitude, and shape characteristics of the brainstem. Lower values of the 90th percentile intensity and energy—indicating fewer highly hyperintense voxels and a lower overall signal magnitude within the region of interest (ROI), respectively—were associated with higher model predictions for PSP. Moreover, lower sphericity, suggesting a more irregular brainstem shape, was also linked to an increased predicted probability of PSP. As illustrated in Fig. 2, the top‐ranked feature showed two clusters of contributions, with lower values increasing the predicted probability and higher values decreasing it, suggesting a strong discriminative role of this feature. Energy and sphericity also showed a wider range of SHAP values, with lower values contributing more strongly to the prediction, whereas higher values had a smaller impact. Importantly, several influential features identified by SHAP remained largely consistent across additional analyses, such as models trained using FreeSurfer‐derived segmentations (Fig. S3) and the reversed‐cohort experiment (Fig. S4), further supporting the stability and robustness of the radiomics‐derived signature. Our findings suggest that radiomic biomarkers—even when applied to standard T1‐weighted images—may offer a powerful and fully automated approach to improve early differential diagnosis in the neurodegenerative field. This is particularly relevant given the growing interest in disease‐modifying therapies for PSP, where timely and accurate classification is crucial for inclusion in clinical trials.^13^ Future work could expand this framework to other regions implicated in atypical parkinsonism, or integrate multimodal MRI acquisitions—including QSM, neuromelanin‐sensitive imaging, or diffusion metrics—to further enhance model performance or explore classification tasks in PSP or PD subgroups. Among the methodological strengths of this study, we highlight the inclusion of two large independent patient cohorts, involving an international multicenter cohort derived from the PPMI and 4RTNI initiatives, which allowed us to validate the findings and to support the generalizability and real‐world applicability of the proposed radiomics approach. We additionally evaluated all models using brainstem masks derived from two independent segmentation pipelines—FreeSurfer and AssemblyNet—and observed highly consistent performance, demonstrating that the radiomics‐derived signatures were robust to the choice of segmentation method. Nonetheless, several limitations should be acknowledged. First, although widely accepted international diagnostic criteria were used, the lack of pathological confirmation remains an important limitation to be acknowledged. Second, our cohort included only the classical PSP‐RS phenotype, and the performance of the proposed biomarker across other PSP variants remains to be established. Third, although the sample size was relatively large and externally validated, future prospective studies with more heterogeneous cohorts, including patients with rarer disease forms or uncertain diagnosis, are needed to evaluate robustness in real‐world settings. Fourth, it should be noted that PSP and PD patients in the validation cohort were drawn from two separate international initiatives (4RTNI and PPMI, respectively). Although all cohorts used harmonized 3 T T1‐weighted protocols, residual interstudy variability in scanner models and acquisition parameters cannot be fully excluded. In conclusion, our findings demonstrate that brainstem radiomics derived from standard T1‐weighted MRI provides an accurate and validated automated biomarker for differentiating PSP from PD, at no significant additional cost of standard volumetric measures, offering a promising tool for supporting the clinical diagnosis in patients with parkinsonian syndromes.

## Author Roles

(1) Research Project: A. Conception, B. Organization, C. Execution; (2) Statistical Analysis: A. Design, B. Execution, C. Review and Critique; (3) Manuscript Preparation: A. Writing of the First Draft, B. Review and Critique.

Ch.C.: 1A, 1C, 2A, 2B, 3A.

J.B.: 1B, 3B.

A.A.: 1B, 3B.

Ca.C.: 2B, 2C, 3B.

M.G.B.: 2B, 2C, 3B.

A.S.: 2B, 2C, 3B.

P.P.A.: 1B, 3B.

Al.Q.: 1A, 1B, 2C, 3B.

An.Q.: 1A, 1B, 2A, 2C, 3B.

## Supporting information


**Figure S1** Example of automated segmentation failure in one progressive supranuclear palsy (PSP) subject from the training cohort. The AssemblyNet volumetry report classified the segmentation quality as “C” (poor quality, output not recommended for use). Visual inspection revealed substantial mis‐segmentation and anatomically implausible delineation of brain structures, with volumetric estimates outside the expected normative range. This case was therefore excluded after quality control.

**Figure S2** Comparison of brainstem volume estimates obtained using FreeSurfer and AssemblyNet. Scatter plot showing the relationship between brainstem volumes derived from FreeSurfer and AssemblyNet segmentations across all subjects. The dashed diagonal line represents perfect agreement between the two methods. The subjects showing the largest relative volumetric differences between the two segmentation pipelines are highlighted and reported in the accompanying table, which includes the corresponding volume estimates obtained with each method and the percentage difference between them.

**Figure S3** Global SHapley Additive exPlanations (SHAP) summary plot showing the contribution of each radiomic feature to the Support Vector Machine (SVM) model trained on Freesurfer‐derived brainstem features. Each point represents one patient, with color indicating the feature value (red = high, blue = low). Features are ranked by mean absolute SHAP value, reflecting their overall impact on model output. Radiomic features marked with an asterisk (*) indicate those that were also identified among the most important features in the model derived from AssemblyNet‐based segmentation.

**Figure S4** Global SHapley Additive exPlanations (SHAP) summary plot showing the contribution of each radiomic feature to the XGBoost model obtained in the reversed‐cohort analysis, where models were trained on the pooled Parkinson's Progression Markers Initiative (PPMI) and 4‐Repeat Tauopathy Neuroimaging Initiative (4RTNI) datasets and validated on the independent cohort from our site. Each point represents one patient, with color indicating the feature value (red = high, blue = low). Features are ranked by mean absolute SHAP value, reflecting their overall impact on model output. Radiomic features marked with an asterisk (*) indicate those that were also identified among the most important features in the main analysis (training = Italian; validation = international).

**Figure S5** Sensitivity analysis illustrating the effect of the Pearson correlation threshold for feature selection on classification performance and generalization across all four classifiers. For each threshold value, the training area under the curve (AUC) (solid line, left y‐axis) and validation AUC (dashed line, left y‐axis) are shown, with shaded bands representing ±1 standard deviation across outer folds. Gray bars (right y‐axis) indicate the absolute performance degradation (|Delta AUC| = |training AUC − validation AUC|). The number of retained features (n) is annotated for each threshold value.

**Figure S6** Calibration plots for the validation cohort across all classifiers, showing the relationship between mean predicted probabilities and the observed fraction of positive outcomes across probability bins. The diagonal line represents perfect calibration. Calibration performance was quantified using the Brier score and the Hosmer–Lemeshow goodness‐of‐fit test. The Brier score reflects the mean squared difference between predicted probabilities and observed outcomes (lower values indicate better calibration), while a non‐significant Hosmer–Lemeshow test (*P* > 0.05) indicates no evidence of miscalibration.

Supporting Information S1

**Table S1** Volume‐based and radiomics‐based classification performance using Freesurfer brainstem segmentation

## Data Availability

Data from the Parkinson's Progression Markers Initiative (PPMI) and the 4‐Repeat Tauopathy Neuroimaging Initiative (4RTNI) are publicly available through their respective data access procedures. Due to ethical and privacy restrictions, individual‐level clinical and imaging data from the training cohort are not publicly available but can be accessed from the corresponding author upon reasonable request. The code used for data processing and analysis is available from the corresponding author upon reasonable request.
