# Supplementary material for: A Brainstem Radiomics Framework to Distinguish Progressive Supranuclear Palsy from Parkinson's Disease
Source: Mov Disord. 2026 May 1;41(8):2133–42. doi: 10.1002/mds.70334 (PMC13518260; doi:10.1002/mds.70334)
Supplement: Supplementary file 1 [file MDS-41-2133-s003.docx]

**Supplementary material**

**Supplementary Methods**

*MRI Acquisition protocols*

University “Magna Graecia” of Catanzaro, Italy:

All study participants underwent a brain 3T MRI examination with either a 3T MR750 General Electric scanner (Discovery MR-750, GE, Milwaukee, WI, USA) with an 8-channel head coil (125 PD and 76 PSP) or a hybrid 3T PET-MR scanner (Biograph mMR, Siemens Healthcare, Erlangen, Germany) using a 16-channel PET-transparent head/neck coil (52 PD and 8 PSP), including 3D T1-weighted images. For the MR-750 GE scanner the protocol included a 3D T1-weighted volumetric spoiled gradient echo (BRAVO, sagittal planes; repetition time/echo time 9.2/3.7 milliseconds; voxel size: 1.0 mm; slice thickness: 1.0 mm, frequency and phase encoding matrix 256 x 256; flip angle 12°; field of view 256 mm). For the SIEMENS scanner, the protocol included a 3D T1-weighted magnetization-prepared rapid acquisition gradient-echo sequence (MPRAGE, sagittal planes, phase encoding matrix 256 × 248, voxel size 1.0 mm, slice thickness: 1.0 mm. repetition time/echo time 2300/2.34 milliseconds, flip angle 8°, field of view 256 mm).

4RTNI:

Patients underwent brain MRI with 3T scanners (GE Signa HDxt, GE Discovery MR750 or SIEMENS TrioTim). The imaging protocol included high-resolution three-dimensional T1-weighted images acquired in the sagittal plane using a volumetric MPRAGE sequence (TR ≈ 2300 ms, TE ≈ 3 ms, TI ≈ 900 ms, flip angle ≈ 9°), with near-isotropic voxel resolution (~1 mm³; slice thickness 1.0 mm for Siemens and 1.2 mm for GE scanners) providing whole-brain coverage. Imaging protocols were harmonized across sites within the 4RTNI initiative to minimize inter-scanner variability.

PPMI:

MRI data from the PPMI cohort were acquired across multiple centers using harmonized 3T MRI protocols according to the PPMI standardized acquisition guidelines. All participants underwent a high-resolution 3D T1-weighted structural scan (MPRAGE or equivalent) acquired in the sagittal plane with 1.0 mm isotropic voxels and whole-brain coverage. Typical acquisition parameters included TR ≈ 2300 ms, TE ≈ 2.9 ms, TI ≈ 900 ms, and a flip angle of approximately 9°. Scanner systems included Siemens, GE, and Philips 3T platforms depending on the site. Detailed acquisition parameters for each site and system are publicly available in the PPMI MRI protocol documentation ([www.ppmi-info.org](http://www.ppmi-info.org)).
