# Supplementary material for: A Brainstem Radiomics Framework to Distinguish Progressive Supranuclear Palsy from Parkinson's Disease
Source: Mov Disord. 2026 May 1;41(8):2133–42. doi: 10.1002/mds.70334 (PMC13518260; doi:10.1002/mds.70334)
Supplement: Supplementary file 2 [file MDS-41-2133-s004.docx]

**Supplementary Table 1: Volume-based and radiomics-based classification performance using Freesurfer brainstem segmentation**

| **Algorithm** | **Accuracy (train/val)** | **AUC**  **(train/val)** | **Precision**  **(train/val)** | **Recall or Sensitivity**  **(train/val)** | **Specificity**  **(train/val)** | **F1**  **(train/val)** |
| --- | --- | --- | --- | --- | --- | --- |
| Brainstem volume  Decision Tree  SVM  Random Forest  XGBoost | 0.83 / 0.82  0.83 / 0.80  0.85 / 0.59  0.86 / 0.82  0.88 / 0.85 | 0.89 / 0.87  0.89 / 0.85  0.89 / 0.77  0.92 / 0.89  0.92 / 0.90 | 0.69 / 0.82  0.80 / 0.71  0.84 / 0.49  0.82 / 0.76  0.84 / 0.86 | 0.87 / 0.66  0.66 / 0.81  0.66 / 0.75  0.71 / 0.82  0.77 / 0.75 | 0.81 / 0.91  0.91 / 0.78  0.94 / 0.49  0.93 / 0.83  0.93 / 0.92 | 0.77 / 0.73  0.71 / 0.76  0.73 / 0.59  0.76 / 0.79  0.80 / 0.80 |

Abbreviations: train = training cohort; val = validation cohort; AUC = Area Under the Curve; SVM = Support Vector Machine; XGBoost = eXtreme Gradient Boosting.
